# Graduate Study in London Special Hospitals

**Published:** 1918-09-07

**Authors:** 


### GRADUATE STUDY IN LONDON SPECIAL HOSPITALS.

**Central London Throat and Ear Hospital, Gray's Inn Road, W.C.**—Three courses of instruction are open to practitioners attending the hospital: first, the course in methods of examination and diagnosis; second, the course of systematic instruction in the diseases of the nose, throat, and ear; and third, the operative surgery class. The course in methods of examination and diagnosis is introductory in character. It comprises four lessons of practical teaching in the actual examination of patients and in the manipulation of instruments. Clinical assistants, especially if they have not had any previous experience in the speciality, are expected to attend this class in order to become acquainted with the minutiæ of the methods of diagnosis, etc. The class is free to clinical assistants. To ordinary students and practitioners the fee is one guinea. Intending students (who can join at any time) are requested to give their names to the Dean. Attendance at this class is compulsory for those who desire to obtain the higher-grade certificate. The second course of systematic instruction in diseases is more advanced. It consists of over thirty lessons in all on pathology, diagnosis, and treatment. Minute details in operative surgery are not gone into, as this is left to the operative surgery class. Full syllabus may be had on application to the Secretary.

**Hospital for Consumption and Diseases of the Chest, Brompton, S.W.**—Owing to the war the courses of special lectures usually given during term have been suspended, but the large clinical field of the hospital is still available for those who wish to study the diseases of the chest under the supervision of the physicians or assistant physicians. Students may enter for courses of clinical instruction in the wards or out-patient department at any time. Arrangements can be made for courses of instruction in special laboratory methods. Special courses of instruction in the diagnosis and treatment of pulmonary tuberculosis are held from time to time. For particulars apply to the Dean.

**Hospital for Diseases of the Throat, Golden Square, W.** (with which is amalgamated The London Throat Hospital, Great Portland Street).—Clinical instruction in the diagnosis and treatment of disease is given daily in the out-patient department from 2 to 5 P.M., and on Tuesdays and Fridays from 6.30 to 9 P.M. The hospital contains 60 beds for in-patients. There is an annual out-patient attendance of over 50,000. Minor operations are performed daily (except Monday) at 9.30 A.M. Major operations are performed on Tuesdays, Wednesdays, Thursdays, Fridays, and Saturdays at 10 A.M. Also Fridays at 2 P.M. Practitioners and medical students are admitted to the practice of the hospital at a fee of five guineas for three months, seven guineas for six months, or ten guineas for perpetual studentship. From amongst the students junior clinical assistants are appointed periodically. For terms and further information apply to the Dean, Mr. Geo. W. Dawson.

**Hospital for Sick Children, Great Ormond Street, W.C.**—This school is one of the post-graduate teaching schools of the University of London, and is recognised by the Conjoint Board of England as a place where undergraduates may spend six months of their fifth year in clinical work. There are 210 beds, to which nearly 3,000 cases are admitted per annum, and in the out-patient department the average number of new cases each year exceeds 24,000. Clinical instruction is given daily in the wards, out-patient department, and operating theatre by members of the visiting staff. Fees for one month's attendance, two guineas; for three months, five guineas; perpetual ticket, ten guineas; clinical clerk's reduced fee of £1 1s. for three months. A certificate is given at the end of a three months' course. Further details and full syllabus may be obtained by application to the Dean or the Secretary of the hospital. A limited number of clerkships in the pathological department, affording facilities for theoretical and practical instruction in clinical pathology and bacteriology suitable for post-graduates and fifth-year students, are available. Fees for one month's course, three guineas; two months' course, five guineas; three months' course, six guineas. For detailed syllabus application should be made to the Dean or the Secretary. The Dean is Mr. George Waugh, from whom all further information can be obtained, or from the Secretary at the hospital.

**Hospital for Women, Soho.**—In connection with the out-patient department there has been for some years a well-organised clinical department. To meet the want increasingly felt by medical practitioners of a more intimate knowledge of the diseases peculiar to women, and of more extended opportunities for examining gynæcological cases, clinical assistants are appointed to the honorary medical officers to out-patients. The appointments are open to qualified medical men and women.

The hospital contains sixty-seven beds. In the out-patient department there are 4,000 new cases during the year, the total number of out-patient attendances being from 14,000 to 16,000. This large number affords exceptional opportunities for seeing and studying most of the varieties of the diseases of women. Every facility is afforded clinical assistants by the honorary medical officers in the out-patient department of examining patients and obtaining experience in diagnosis and treatment.

The number of clinical assistants appointed to each gynæcologist is so far as possible limited to two, though occasionally a third may be appointed. The clinical assistants attend on two afternoons a week, and it is advisable that they should take out a three months' course, but to meet the convenience of medical practitioners it is permitted to attend for a shorter period, and on one or more days a week if it can be arranged.

**National Hospital for the Paralysed and Epileptic, Queen Square, W.C.**—The in- and out-patient practice of this hospital is open at 2 P.M. every day (except Wednesday and Saturday). Patients are seen by the physicians, the physicians for out-patients, and the assistant physicians. Three courses of lectures and clinical demonstrations are given in each year. The fee for out-patient hospital practice and lectures is one guinea for three months, three guineas per annum, and five guineas for a perpetual ticket; for in-patient clerking a fee of two guineas for three months or three guineas for six months is charged. The museum is available for the prosecution of special work under the pathologist, for which a separate fee of two guineas for the three months is charged. Women students attend the practice of the hospital.

**Ophthalmic Hospitals.**—Ophthalmic hospitals are the Central London Ophthalmic Hospital, Judd Street, W.C.; the Royal London Ophthalmic Hospital, City Road, E.C.; the Royal Eye Hospital, St. George's Circus, S.E.; and the Royal Westminster Ophthalmic Hospital, King William Street, W.C. In each of these the fees charged are very modest.

**Queen Charlotte's Lying-in Hospital.**—Qualified medical practitioners and medical students are admitted to the practice of this hospital. Last year (1917) there were 1,947 women delivered in the wards, about one-half being primiparæ. A large number of the cases were abnormal. During the present year the number of women delivered in the wards has been at the rate of nearly 2,000 per annum. Certificates of attendance at this hospital are recognised by all Universities, Colleges, and licensing bodies. Fee for the course of four weeks, £8 8s. Students are accommodated at the new Residential College (5 Cosway Street) opposite the hospital.

Arrangements have also been made for the preliminary instruction in midwifery now required by the General Medical Council. This instruction will include: (1) Practical instruction in the methods of examination of pregnant women; (2) delivery of women in labour under the direct supervision of a medical officer of the hospital; (3) practical instruction in the treatment of the mother and child during the puerperium, including clinics held four times weekly by the visiting medical staff; (4) instruction in the clinical laboratory of the hospital. The fee for this special course for students will be £5 5s. for the course of one month.

Eighty-eight students (sixty men and twenty-eight women) and eighteen qualified practitioners (sixteen men and two women) attended the practice of the hospital during 1917.

**Royal Hospital for Diseases of the Chest City Road, E.C.**—This hospital, which has lately undergone extensive reorganisation, provides an excellent field for research and study in diseases of the chest, more especially in tuberculosis in its various departments. The departments in which study can be pursued comprise the in-patient, out-patient, Röntgen-ray, cardiological, pathological and bacteriological, ear, nose and throat, and dental departments. An extensive department for the prevention of consumption (tuberculosis dispensary) has been established for nearly four years. As this works in connection with three large boroughs—namely, Shoreditch, Finsbury, and Islington—a splendid opportunity presents itself for medical practitioners and students who desire to obtain an intimate knowledge of this work. The bacteriological and pathological research laboratories, under the new director, Dr. Carnegie Dickson, should provide excellent opportunities for acquiring a knowledge of bacteriological methods, and also a field for research for those who wish to pursue such studies. The cardiological department, which has recently been established, has been equipped with all the latest apparatus for the investigation of diseases of the heart. In connection with the Röntgen ray department, there are two radiologists, who attend the hospital four days weekly. Attached to the hospital is a medical school for instruction in diseases of the chest, more especially tuberculosis, at which courses of instruction are given from time to time. There is also a tuberculosis school for nurses, where regular theoretical and thoroughly practical courses of instruction are given, for which a certificate is granted. Owing to several of the staff being engaged on "war work," it has been found impossible to arrange a theoretical course of lectures, but the clinical practice of the hospital will be open to medical practitioners and students as usual. The fees for attending the clinical practice of the hospital are as follows: One month, one guinea; three months, three guineas; six months, five guineas; perpetual ticket, six guineas. Any further information will be gladly given by the Dean, Major Barty King.

**St. John's Hospital for Diseases of the Skin.**—Out-patient Department, 49 Leicester Square, W.C. 2; In-patient Department, 262 Uxbridge Road, W. This hospital has a well-equipped in-patient department, with 40 beds. It has a School of Dermatology at 49 Leicester Square, which is conducted by the medical staff of the hospital. During the winter the free course of Chesterfield lectures is given by Dr. Morgan Dockrell, and is always well attended by the profession. Other free courses are given by Capt. W. Griffith, Dr. J. L. Bunch, and Dr. Knowsley Sibley. The out-patient department contains a spacious laboratory and an electrical department. Special courses in the pathology and bacteriology of the skin may be arranged for. The daily clinics at 2, and every evening (except Saturday) at 6, are open to practitioners, upon signing the Visitors' Book. Venereal diseases, under the Government scheme, are seen at all clinics.

